# *N*-*tert*-Butyl-3-hydr­oxy-5-androstene-17-carboxamide monohydrate

**DOI:** 10.1107/S1600536809020984

**Published:** 2009-06-06

**Authors:** Jiang-Sheng Li, Jim Simpson, Xiao-Jun Li, Xun Li, Peng-Mian Huang

**Affiliations:** a School of Chemistry and Biological Engineering, Changsha University of Science & Technology, Changsha 410004, People’s Republic of China; b Department of Chemistry, University of Otago, PO Box 56, Dunedin, New Zealand; c School of Chemical Engineering, Hebei University of Technology, Tianjin 300130, People’s Republic of China

## Abstract

In the title compound, C_24_H_39_NO_2_·H_2_O, the *A* and *C* rings of the pregnolene derivative sterol adopt chair conformations, with the *B* ring in a flattened chair conformation and the five-membered ring in an envelope conformation twisted about the *C*/*D* ring junction. The *N*-*tert*-butyl­carboxamide substituent is equatorial. The 3β-hydr­oxy H atom and one H atom of the water mol­ecule are disordered over two positions with equal occupancies. In the crystal structure, O—H⋯O hydrogen bonds between the 3β-hydr­oxy groups of neighbouring mol­ecules form dimers in the *bc* plane and these dimers are stacked along the *a* axis by additional O—H⋯O hydrogen bonds involving the water mol­ecules. The steric effect of the bulky *tert*-butyl substituent in the carboxamide chain precludes hydrogen-bond formation by the N—H group.

## Related literature

The title compound is an inter­mediate in the synthesis of finasteride (Li *et al.*, 2001 ▶). For pharmaceutical applications of finasteride, systematic name *N*-(*tert*-but­yl)-3-oxo-4-aza-5-androst-1-ene-17-carboxamide, see: Rasmusson *et al.* (1984 ▶, 1986 ▶); Rasmusson & Reynold (1985 ▶); US National Library of Medicine and National Institutes of Health (2008 ▶). For pregnenolone and its derivatives, see: Finar (1959 ▶). For the preparation of the title compound, see: Rasmusson *et al.* (1984 ▶); Dolling *et al.* (1999 ▶). For related structures, see: Bordner *et al.* (1978 ▶); Lancaster *et al.* (2007 ▶); Duax *et al.* (1989 ▶); Shukla *et al.* (2008 ▶). For ring puckering analysis, see: Cremer & Pople (1975 ▶). For a description of the Cambridge Structural Database, see: Allen (2002 ▶).
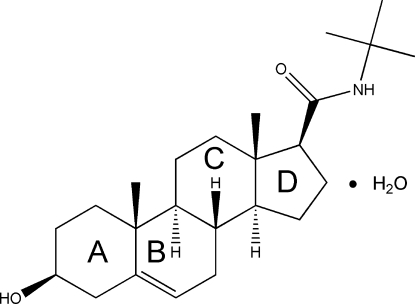


## Experimental

### 

#### Crystal data

C_24_H_39_NO_2_·H_2_O
*M* *_r_* = 391.58
Monoclinic, 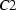

*a* = 9.934 (6) Å
*b* = 7.469 (5) Å
*c* = 30.647 (18) Å
β = 91.547 (10)°
*V* = 2273 (2) Å^3^
*Z* = 4
Mo *K*α radiation
μ = 0.07 mm^−1^
*T* = 293 K
0.40 × 0.30 × 0.15 mm

#### Data collection

Bruker SMART 1K CCD diffractometer
Absorption correction: multi-scan (*SADABS*; Sheldrick, 1996 ▶) *T*_min_ = 0.971, *T*_max_ = 0.989
4186 measured reflections
2146 independent reflections
1735 reflections with *I* > 2σ(*I*)
*R*_int_ = 0.035

#### Refinement

*R*[*F* ^2^ > 2σ(*F* ^2^)] = 0.048
*wR*(*F* ^2^) = 0.114
*S* = 1.07
2146 reflections
277 parameters
7 restraints
H atoms treated by a mixture of independent and constrained refinement
Δρ_max_ = 0.18 e Å^−3^
Δρ_min_ = −0.15 e Å^−3^

### 

Data collection: *SMART* (Bruker, 1997 ▶); cell refinement: *SAINT* (Bruker, 1997 ▶); data reduction: *SAINT*; program(s) used to solve structure: *SHELXS97* (Sheldrick, 2008 ▶); program(s) used to refine structure: *SHELXL97* (Sheldrick, 2008 ▶); molecular graphics: *SHELXTL* (Sheldrick, 2008 ▶) and *Mercury* (Macrae *et al.*, 2006 ▶); software used to prepare material for publication: *SHELXL97*, *enCIFer* (Allen *et al.*, 2004 ▶), *PLATON* (Spek, 2009 ▶) and *publCIF* (Westrip, 2009 ▶).

## Supplementary Material

Crystal structure: contains datablocks global, I. DOI: 10.1107/S1600536809020984/wm2238sup1.cif

Structure factors: contains datablocks I. DOI: 10.1107/S1600536809020984/wm2238Isup2.hkl

Additional supplementary materials:  crystallographic information; 3D view; checkCIF report

## Figures and Tables

**Table 1:** Hydrogen-bond geometry (Å, °)

*D*—H⋯*A*	*D*—H	H⋯*A*	*D*⋯*A*	*D*—H⋯*A*
O1—H1*OA*⋯O1^i^	0.821 (11)	1.99 (4)	2.783 (6)	161 (10)
O1—H1*OB*⋯O1*W*^ii^	0.819 (11)	2.31 (9)	2.756 (5)	115 (8)
O1*W*—H1*W*⋯O1*W*^i^	0.847 (11)	2.26 (6)	2.798 (9)	121 (6)
O1*W*—H2*WA*⋯O1^iii^	0.850 (11)	1.93 (6)	2.756 (5)	163 (20)

Symmetry codes: (i) 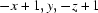; (ii) 
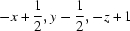
; (iii) 
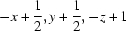
.

**Fig. 1.**
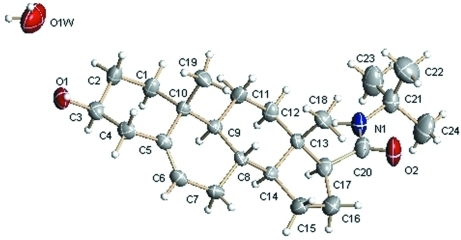
View of the molecular structure of (I) showing the atom-labelling scheme. Displacement ellipsoids are drawn at the 50% probability level and H atoms are shown as small spheres of arbitrary radii. Only one disorder component of the disordered O—H group and water molecule is shown.

**Fig. 2.**
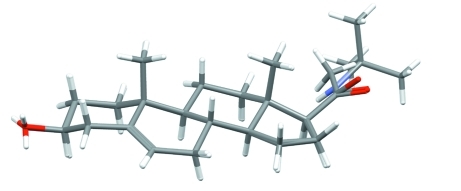
An overlay in Mercury (Macrae et al., 2006) of the non-hydrogen atoms common to the structures of (I) and pregnenolone (Lancaster et al., 2007).

**Fig. 3.**
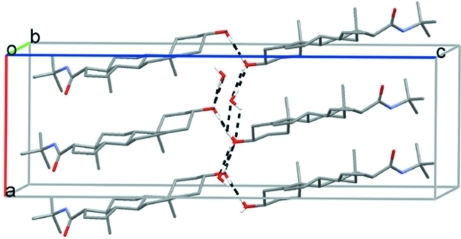
Crystal packing of (I) viewed approximately down the b axis. Hydrogen bonds are drawn as dashed lines and H atoms not involved in hydrogen bonding have been omitted.

## supplementary crystallographic information

### Comment

Finasteride, systematic name *N*-(*tert*-butyl)-3-oxo-4-aza-5-androst-1-ene-17-carboxamide, is an azasteroid used in the treatment of both Benign Prostatic Hypertrophy and hair loss in the male adults (Rasmusson *et al.*, 1984,1986; Rasmusson & Reynold, 1985; US National Library of Medicine and National Institutes of Health, 2008). The title compound, (I), Fig. 1, is a derivative of pregnenolone (Finar, 1959) which crystallizes with a solvent water molecule and is a key intermediate in the synthesis of finasteride (Li *et al.*, 2001).

The A and C rings of the steroid skeleton (C1—C5, C10 and C8,C9,C11—C14) have chair conformations (Cremer & Pople, 1975) with atoms C3 and C10 - 0.668 (5) and 0.604 (5) Å from the C1,C2,C4,C5 plane (r.m.s. deviation 0.018 Å) and atoms C9 and C13 - 0.601 (4) and 0.701 (4) Å from the C8,C11,C12,C14 plane (r.m.s. deviation 0.011 Å). Ring B (C5—C10) has a flattened chair conformation with an r.m.s. deviation of 0.091 Å from the plane through atoms C5—C7,C9,C10 with atom C8 displaced 0.588 (4) Å from this plane. The five membered ring D (C13—C17) has an envelope conformation twisted about the C/D ring junction with atom C14 - 0.617 (5) Å from the plane through C13,C15—C17 (r.m.s. deviation 0.082 Å).

The close similarity between this molecule and pregnenolone is amply demonstrated by the fact that the structure overlays with that of pregnenolone (Lancaster *et al.*, 2007; refcode PREGOL01 in the Cambridge Structural Database, version 5.30, Feb. 2009; Allen, 2002) with an r.m.s. deviation of 0.111 Å. This deviation drops to 0.045 Å if the carbonyl O2 atom is omitted from the calculation, Fig. 2 (Macrae *et al.*, 2006). The C16—C17—C20—O2 torsion angle which defines the conformation of the carboxamide group with respect to the main skeleton is -26.9 (5) °, significantly greater than the values in the range -3.0 to 0.6 ° reported for pregnenolone itself (Bordner *et al.*, 1978; Lancaster *et al.*, 2007) but more comparable to those found in pregnenolone derivatives with substituents elsewhere on the steroid backbone (Duax *et al.*, 1989; Shukla *et al.*, 2008).

In the crystal structure O1—H1OA···O1 hydrogen bonds form dimers in the *bc* plane. O1W—H1W···O1W hydrogen bonds link pairs of water molecules, while O1—H1O···O1W and O1W—H2W···O1 interactions stack the dimers along *a* (Fig. 3). The amide N1—H1 group is not involved in hydrogen bonding interactions, presumably because of the shielding effect of the bulky *tert*-butyl substituents.

### Experimental

The title compound was prepared from pregnenolone as previously described (Rasmusson *et al.*, 1984; Dolling *et al.*, 1999). A single-crystal of the title compound, suitable for X-ray analysis, was grown by slow evaporation of the solvent DMF/H2O (1:2 v:v).

### Refinement

The H atoms bound to C were positioned geometrically and constrained to ride on their parent atoms [C—H distances are 0.97–0.98Å for CH_2_ and CH groups with *U*_iso_(H) = 1.2 *U*_eq_(C), and 0.96 Å for CH_3_ groups. The O—H and N—H hydrogen atoms were refined with their isotropic displacement parameters 1.2 *U*_eq_(O,*N*) for the O—H and N—H groups and 1.5 *U*_eq_(O) for the water molecule. Distances were constrained to 0.82 (1) Å for the O—H bond 0.85 (1) Å in the water molecule and 0.90 (1)Å for the N—H bond. The H1O atom on O1 and the H2W atom of the water molecule are each disordered over two positions with equal occupancies. This disorder leads to a close approach of the O1 atoms and their hydrogen atoms on adjacent molecules. The absolute configuration of (I) could not be determined and Friedel equivalents were averaged in the refinement. The chosen configuration was based on that of the pregnenolone precursor with C3 *S*, C8 *R*, C9 *S*, C10 *R*, C13 *S* and C14 *S*.

### Crystal data

**Table:**

C_24_H_39_NO_2_·H_2_O	*F*(000) = 864
*M**_r_* = 391.58	*D*_x_ = 1.144 Mg m^−^^3^
Monoclinic, *C*2	Mo *K*α radiation, λ = 0.71073 Å
Hall symbol: C 2y	Cell parameters from 734 reflections
*a* = 9.934 (6) Å	θ = 3.3–26.2°
*b* = 7.469 (5) Å	µ = 0.07 mm^−^^1^
*c* = 30.647 (18) Å	*T* = 293 K
β = 91.547 (10)°	Block, colourless
*V* = 2273 (2) Å^3^	0.40 × 0.30 × 0.15 mm
*Z* = 4	

### Data collection

**Table:**

Bruker SMART 1K CCD diffractometer	2146 independent reflections
Radiation source: fine-focus sealed tube	1735 reflections with *I* > 2σ(*I*)
graphite	*R*_int_ = 0.035
φ and ω scans	θ_max_ = 25.0°, θ_min_ = 2.0°
Absorption correction: multi-scan (*SADABS*; Sheldrick, 1996)	*h* = −11→11
*T*_min_ = 0.971, *T*_max_ = 0.989	*k* = −5→8
4186 measured reflections	*l* = −32→36

### Refinement

**Table:**

Refinement on *F*^2^	Secondary atom site location: difference Fourier map
Least-squares matrix: full	Hydrogen site location: inferred from neighbouring sites
*R*[*F*^2^ > 2σ(*F*^2^)] = 0.048	H atoms treated by a mixture of independent and constrained refinement
*wR*(*F*^2^) = 0.114	*w* = 1/[σ^2^(*F*_o_^2^) + (0.0584*P*)^2^ + 0.5179*P*] where *P* = (*F*_o_^2^ + 2*F*_c_^2^)/3
*S* = 1.07	(Δ/σ)_max_ = 0.003
2146 reflections	Δρ_max_ = 0.18 e Å^−^^3^
277 parameters	Δρ_min_ = −0.15 e Å^−^^3^
7 restraints	Extinction correction: *SHELXL97* (Sheldrick, 2008), Fc^*^=kFc[1+0.001xFc^2^λ^3^/sin(2θ)]^-1/4^
Primary atom site location: structure-invariant direct methods	Extinction coefficient: 0.0095 (13)

### Special details

**Table:**

Geometry. All e.s.d.'s (except the e.s.d. in the dihedral angle between two l.s. planes) are estimated using the full covariance matrix. The cell e.s.d.'s are taken into account individually in the estimation of e.s.d.'s in distances, angles and torsion angles; correlations between e.s.d.'s in cell parameters are only used when they are defined by crystal symmetry. An approximate (isotropic) treatment of cell e.s.d.'s is used for estimating e.s.d.'s involving l.s. planes.
Refinement. Refinement of *F*^2^ against ALL reflections. The weighted *R*-factor *wR* and goodness of fit *S* are based on *F*^2^, conventional *R*-factors *R* are based on *F*, with *F* set to zero for negative *F*^2^. The threshold expression of *F*^2^ > σ(*F*^2^) is used only for calculating *R*-factors(gt) *etc*. and is not relevant to the choice of reflections for refinement. *R*-factors based on *F*^2^ are statistically about twice as large as those based on *F*, and *R*- factors based on ALL data will be even larger.

### Fractional atomic coordinates and isotropic or equivalent isotropic displacement parameters (Å^2^)

**Table:**

	*x*	*y*	*z*	*U*_iso_*/*U*_eq_	Occ. (<1)
C1	0.4428 (4)	0.2304 (5)	0.35592 (10)	0.0384 (9)	
H1A	0.4627	0.3396	0.3403	0.046*	
H1B	0.3577	0.1847	0.3443	0.046*	
C3	0.3985 (3)	0.1094 (5)	0.42947 (10)	0.0379 (9)	
H3	0.3143	0.0565	0.4181	0.045*	
O1	0.3816 (3)	0.1542 (4)	0.47468 (8)	0.0467 (7)	
H1OA	0.450 (6)	0.128 (13)	0.489 (3)	0.070*	0.50
H1OB	0.358 (10)	0.052 (5)	0.480 (3)	0.070*	0.50
C4	0.5102 (4)	−0.0249 (5)	0.42418 (11)	0.0374 (9)	
H4A	0.5926	0.0222	0.4374	0.045*	
H4B	0.4877	−0.1344	0.4394	0.045*	
C5	0.5333 (3)	−0.0670 (5)	0.37715 (10)	0.0300 (8)	
C6	0.5374 (3)	−0.2341 (5)	0.36342 (11)	0.0350 (8)	
H6	0.5238	−0.3239	0.3838	0.042*	
C7	0.5621 (3)	−0.2899 (5)	0.31783 (11)	0.0346 (8)	
H7A	0.4788	−0.3350	0.3048	0.042*	
H7B	0.6269	−0.3871	0.3183	0.042*	
C8	0.6145 (3)	−0.1399 (5)	0.28958 (10)	0.0273 (7)	
H8	0.7102	−0.1216	0.2968	0.033*	
C9	0.5391 (3)	0.0331 (4)	0.29865 (10)	0.0264 (7)	
H9	0.4435	0.0065	0.2933	0.032*	
C10	0.5527 (3)	0.0933 (4)	0.34704 (10)	0.0268 (7)	
C19	0.6913 (3)	0.1751 (5)	0.35760 (10)	0.0388 (9)	
H19A	0.7037	0.1859	0.3887	0.058*	
H19B	0.6969	0.2914	0.3444	0.058*	
H19C	0.7602	0.0992	0.3463	0.058*	
C11	0.5739 (4)	0.1816 (5)	0.26685 (10)	0.0365 (8)	
H11A	0.6649	0.2223	0.2735	0.044*	
H11B	0.5138	0.2819	0.2713	0.044*	
C12	0.5642 (4)	0.1268 (5)	0.21887 (10)	0.0372 (9)	
H12A	0.4708	0.1039	0.2106	0.045*	
H12B	0.5963	0.2239	0.2009	0.045*	
C13	0.6473 (3)	−0.0400 (5)	0.21091 (10)	0.0292 (8)	
C18	0.7972 (3)	0.0009 (5)	0.21675 (12)	0.0404 (9)	
H18A	0.8486	−0.1026	0.2090	0.061*	
H18B	0.8169	0.0317	0.2467	0.061*	
H18C	0.8204	0.0994	0.1983	0.061*	
C14	0.5993 (3)	−0.1847 (4)	0.24156 (10)	0.0288 (8)	
H14	0.5022	−0.1957	0.2357	0.035*	
C15	0.6607 (4)	−0.3561 (5)	0.22482 (12)	0.0451 (10)	
H15A	0.6081	−0.4594	0.2331	0.054*	
H15B	0.7524	−0.3708	0.2360	0.054*	
C16	0.6573 (4)	−0.3311 (6)	0.17535 (12)	0.0553 (11)	
H16A	0.5903	−0.4094	0.1618	0.066*	
H16B	0.7444	−0.3592	0.1635	0.066*	
C17	0.6212 (4)	−0.1340 (6)	0.16646 (11)	0.0389 (9)	
H17	0.5244	−0.1277	0.1596	0.047*	
C20	0.6943 (4)	−0.0543 (6)	0.12881 (12)	0.0448 (10)	
O2	0.8041 (3)	−0.1105 (5)	0.11812 (9)	0.0679 (10)	
N1	0.6348 (3)	0.0865 (5)	0.10950 (9)	0.0459 (9)	
H1N	0.553 (2)	0.120 (6)	0.1186 (12)	0.055*	
C21	0.6855 (4)	0.1911 (7)	0.07302 (11)	0.0499 (11)	
C22	0.8179 (5)	0.2794 (9)	0.08648 (17)	0.0811 (16)	
H22A	0.8044	0.3557	0.1112	0.122*	
H22B	0.8501	0.3494	0.0626	0.122*	
H22C	0.8830	0.1889	0.0941	0.122*	
C23	0.5815 (5)	0.3306 (8)	0.06214 (15)	0.0794 (17)	
H23A	0.4980	0.2733	0.0540	0.119*	
H23B	0.6112	0.4024	0.0383	0.119*	
H23C	0.5686	0.4054	0.0871	0.119*	
C24	0.7039 (5)	0.0700 (9)	0.03397 (13)	0.0816 (17)	
H24A	0.7758	−0.0129	0.0402	0.122*	
H24B	0.7255	0.1410	0.0090	0.122*	
H24C	0.6221	0.0050	0.0280	0.122*	
O1W	0.3597 (4)	0.5303 (7)	0.49476 (16)	0.1038 (14)	
H1W	0.416 (6)	0.553 (13)	0.5152 (16)	0.156*	
H2WA	0.288 (15)	0.55 (3)	0.508 (7)	0.156*	0.50
H2WB	0.299 (18)	0.60 (2)	0.503 (8)	0.156*	0.50
C2	0.4264 (4)	0.2760 (5)	0.40398 (11)	0.0408 (9)	
H2A	0.5080	0.3323	0.4154	0.049*	
H2B	0.3527	0.3599	0.4070	0.049*	

### Atomic displacement parameters (Å^2^)

**Table:**

	*U*^11^	*U*^22^	*U*^33^	*U*^12^	*U*^13^	*U*^23^
C1	0.049 (2)	0.037 (2)	0.0293 (18)	0.0108 (17)	0.0050 (15)	0.0067 (17)
C3	0.0364 (19)	0.046 (2)	0.0312 (18)	−0.0048 (18)	−0.0004 (13)	−0.0004 (17)
O1	0.0488 (17)	0.061 (2)	0.0309 (14)	−0.0033 (15)	0.0086 (11)	−0.0015 (14)
C4	0.048 (2)	0.036 (2)	0.0277 (18)	−0.0011 (17)	0.0005 (15)	0.0079 (16)
C5	0.0281 (17)	0.032 (2)	0.0299 (17)	−0.0031 (15)	−0.0014 (13)	0.0066 (16)
C6	0.038 (2)	0.033 (2)	0.0345 (19)	−0.0038 (16)	0.0023 (14)	0.0095 (17)
C7	0.0400 (19)	0.0231 (19)	0.041 (2)	−0.0042 (15)	0.0028 (15)	0.0031 (16)
C8	0.0254 (16)	0.0282 (18)	0.0286 (17)	−0.0008 (15)	0.0034 (12)	0.0023 (15)
C9	0.0237 (15)	0.0247 (17)	0.0306 (17)	0.0005 (13)	0.0002 (12)	0.0023 (14)
C10	0.0296 (17)	0.0271 (18)	0.0237 (16)	0.0045 (14)	0.0023 (12)	0.0027 (14)
C19	0.0403 (19)	0.043 (2)	0.0332 (18)	−0.0118 (18)	0.0021 (14)	0.0019 (18)
C11	0.054 (2)	0.0279 (19)	0.0284 (18)	0.0077 (18)	0.0066 (15)	0.0042 (16)
C12	0.043 (2)	0.041 (2)	0.0278 (18)	0.0085 (17)	0.0038 (14)	0.0069 (17)
C13	0.0269 (17)	0.032 (2)	0.0288 (18)	−0.0008 (15)	0.0037 (13)	−0.0001 (16)
C18	0.0337 (19)	0.044 (2)	0.044 (2)	−0.0035 (17)	0.0026 (15)	0.0073 (18)
C14	0.0256 (16)	0.0274 (19)	0.0335 (18)	−0.0029 (14)	0.0020 (13)	−0.0041 (15)
C15	0.056 (2)	0.036 (2)	0.043 (2)	−0.0040 (19)	0.0084 (17)	−0.0065 (18)
C16	0.068 (3)	0.051 (3)	0.048 (2)	−0.007 (2)	0.0149 (19)	−0.014 (2)
C17	0.0376 (19)	0.049 (2)	0.0301 (18)	−0.0050 (18)	0.0024 (14)	−0.0062 (18)
C20	0.044 (2)	0.057 (3)	0.033 (2)	−0.001 (2)	0.0091 (16)	−0.007 (2)
O2	0.062 (2)	0.089 (3)	0.0546 (18)	0.0252 (18)	0.0296 (14)	0.0116 (18)
N1	0.0422 (17)	0.064 (2)	0.0315 (16)	0.0094 (17)	0.0086 (13)	0.0049 (17)
C21	0.052 (2)	0.066 (3)	0.032 (2)	0.009 (2)	0.0086 (16)	0.004 (2)
C22	0.068 (3)	0.096 (4)	0.079 (3)	−0.017 (3)	0.000 (3)	0.010 (3)
C23	0.088 (3)	0.095 (4)	0.056 (3)	0.027 (3)	0.019 (2)	0.021 (3)
C24	0.106 (4)	0.100 (5)	0.039 (2)	0.021 (4)	0.013 (2)	−0.005 (3)
O1W	0.076 (3)	0.106 (4)	0.131 (4)	0.021 (3)	0.019 (2)	−0.036 (3)
C2	0.049 (2)	0.041 (2)	0.0326 (19)	0.0135 (18)	0.0085 (15)	−0.0001 (18)

### Geometric parameters (Å, °)

**Table:**

C1—C2	1.525 (5)	C13—C18	1.526 (5)
C1—C10	1.527 (5)	C13—C17	1.548 (5)
C1—H1A	0.9700	C18—H18A	0.9600
C1—H1B	0.9700	C18—H18B	0.9600
C3—O1	1.440 (4)	C18—H18C	0.9600
C3—C2	1.499 (5)	C14—C15	1.514 (5)
C3—C4	1.508 (5)	C14—H14	0.9800
C3—H3	0.9800	C15—C16	1.527 (5)
O1—H1OA	0.821 (11)	C15—H15A	0.9700
O1—H1OB	0.819 (11)	C15—H15B	0.9700
C4—C5	1.499 (5)	C16—C17	1.537 (6)
C4—H4A	0.9700	C16—H16A	0.9700
C4—H4B	0.9700	C16—H16B	0.9700
C5—C6	1.318 (5)	C17—C20	1.503 (5)
C5—C10	1.527 (5)	C17—H17	0.9800
C6—C7	1.485 (5)	C20—O2	1.222 (4)
C6—H6	0.9300	C20—N1	1.337 (5)
C7—C8	1.517 (5)	N1—C21	1.465 (5)
C7—H7A	0.9700	N1—H1N	0.899 (10)
C7—H7B	0.9700	C21—C23	1.499 (6)
C8—C14	1.513 (4)	C21—C24	1.515 (6)
C8—C9	1.522 (5)	C21—C22	1.518 (6)
C8—H8	0.9800	C22—H22A	0.9600
C9—C11	1.523 (5)	C22—H22B	0.9600
C9—C10	1.552 (4)	C22—H22C	0.9600
C9—H9	0.9800	C23—H23A	0.9600
C10—C19	1.533 (4)	C23—H23B	0.9600
C19—H19A	0.9600	C23—H23C	0.9600
C19—H19B	0.9600	C24—H24A	0.9600
C19—H19C	0.9600	C24—H24B	0.9600
C11—C12	1.527 (4)	C24—H24C	0.9600
C11—H11A	0.9700	O1W—H1W	0.847 (11)
C11—H11B	0.9700	O1W—H2WA	0.850 (11)
C12—C13	1.519 (5)	O1W—H2WB	0.850 (11)
C12—H12A	0.9700	C2—H2A	0.9700
C12—H12B	0.9700	C2—H2B	0.9700
C13—C14	1.517 (5)		
			
C2—C1—C10	114.7 (3)	C14—C13—C17	100.0 (3)
C2—C1—H1A	108.6	C12—C13—C17	115.8 (3)
C10—C1—H1A	108.6	C18—C13—C17	109.5 (3)
C2—C1—H1B	108.6	C13—C18—H18A	109.5
C10—C1—H1B	108.6	C13—C18—H18B	109.5
H1A—C1—H1B	107.6	H18A—C18—H18B	109.5
O1—C3—C2	109.6 (3)	C13—C18—H18C	109.5
O1—C3—C4	111.3 (3)	H18A—C18—H18C	109.5
C2—C3—C4	110.4 (3)	H18B—C18—H18C	109.5
O1—C3—H3	108.5	C8—C14—C15	119.1 (3)
C2—C3—H3	108.5	C8—C14—C13	114.8 (3)
C4—C3—H3	108.5	C15—C14—C13	104.9 (3)
C3—O1—H1OA	110 (7)	C8—C14—H14	105.7
C3—O1—H1OB	92 (7)	C15—C14—H14	105.7
H1OA—O1—H1OB	85 (9)	C13—C14—H14	105.7
C5—C4—C3	112.0 (3)	C14—C15—C16	103.6 (3)
C5—C4—H4A	109.2	C14—C15—H15A	111.0
C3—C4—H4A	109.2	C16—C15—H15A	111.0
C5—C4—H4B	109.2	C14—C15—H15B	111.0
C3—C4—H4B	109.2	C16—C15—H15B	111.0
H4A—C4—H4B	107.9	H15A—C15—H15B	109.0
C6—C5—C4	120.8 (3)	C15—C16—C17	107.0 (3)
C6—C5—C10	122.9 (3)	C15—C16—H16A	110.3
C4—C5—C10	116.2 (3)	C17—C16—H16A	110.3
C5—C6—C7	125.0 (3)	C15—C16—H16B	110.3
C5—C6—H6	117.5	C17—C16—H16B	110.3
C7—C6—H6	117.5	H16A—C16—H16B	108.6
C6—C7—C8	113.4 (3)	C20—C17—C16	113.6 (3)
C6—C7—H7A	108.9	C20—C17—C13	115.0 (3)
C8—C7—H7A	108.9	C16—C17—C13	104.2 (3)
C6—C7—H7B	108.9	C20—C17—H17	107.9
C8—C7—H7B	108.9	C16—C17—H17	107.9
H7A—C7—H7B	107.7	C13—C17—H17	107.9
C14—C8—C7	111.4 (3)	O2—C20—N1	122.6 (4)
C14—C8—C9	109.2 (3)	O2—C20—C17	121.5 (4)
C7—C8—C9	110.2 (2)	N1—C20—C17	115.8 (3)
C14—C8—H8	108.7	C20—N1—C21	126.9 (3)
C7—C8—H8	108.7	C20—N1—H1N	118 (3)
C9—C8—H8	108.7	C21—N1—H1N	115 (3)
C8—C9—C11	112.3 (2)	N1—C21—C23	106.9 (3)
C8—C9—C10	112.9 (3)	N1—C21—C24	109.6 (4)
C11—C9—C10	112.6 (3)	C23—C21—C24	109.7 (4)
C8—C9—H9	106.1	N1—C21—C22	109.7 (3)
C11—C9—H9	106.1	C23—C21—C22	110.2 (5)
C10—C9—H9	106.1	C24—C21—C22	110.6 (4)
C5—C10—C1	108.4 (2)	C21—C22—H22A	109.5
C5—C10—C19	108.2 (3)	C21—C22—H22B	109.5
C1—C10—C19	109.8 (3)	H22A—C22—H22B	109.5
C5—C10—C9	109.9 (3)	C21—C22—H22C	109.5
C1—C10—C9	108.7 (2)	H22A—C22—H22C	109.5
C19—C10—C9	111.8 (2)	H22B—C22—H22C	109.5
C10—C19—H19A	109.5	C21—C23—H23A	109.5
C10—C19—H19B	109.5	C21—C23—H23B	109.5
H19A—C19—H19B	109.5	H23A—C23—H23B	109.5
C10—C19—H19C	109.5	C21—C23—H23C	109.5
H19A—C19—H19C	109.5	H23A—C23—H23C	109.5
H19B—C19—H19C	109.5	H23B—C23—H23C	109.5
C9—C11—C12	114.3 (3)	C21—C24—H24A	109.5
C9—C11—H11A	108.7	C21—C24—H24B	109.5
C12—C11—H11A	108.7	H24A—C24—H24B	109.5
C9—C11—H11B	108.7	C21—C24—H24C	109.5
C12—C11—H11B	108.7	H24A—C24—H24C	109.5
H11A—C11—H11B	107.6	H24B—C24—H24C	109.5
C13—C12—C11	110.7 (3)	H1W—O1W—H2WA	98 (10)
C13—C12—H12A	109.5	H1W—O1W—H2WB	96 (10)
C11—C12—H12A	109.5	H2WA—O1W—H2WB	29 (10)
C13—C12—H12B	109.5	C3—C2—C1	110.1 (3)
C11—C12—H12B	109.5	C3—C2—H2A	109.6
H12A—C12—H12B	108.1	C1—C2—H2A	109.6
C14—C13—C12	107.7 (2)	C3—C2—H2B	109.6
C14—C13—C18	113.1 (3)	C1—C2—H2B	109.6
C12—C13—C18	110.5 (3)	H2A—C2—H2B	108.2
			
O1—C3—C4—C5	−177.4 (3)	C7—C8—C14—C15	−55.4 (4)
C2—C3—C4—C5	−55.4 (4)	C9—C8—C14—C15	−177.3 (3)
C3—C4—C5—C6	−129.1 (4)	C7—C8—C14—C13	179.1 (3)
C3—C4—C5—C10	51.6 (4)	C9—C8—C14—C13	57.1 (3)
C4—C5—C6—C7	−178.8 (3)	C12—C13—C14—C8	−60.6 (4)
C10—C5—C6—C7	0.5 (5)	C18—C13—C14—C8	61.8 (4)
C5—C6—C7—C8	13.3 (5)	C17—C13—C14—C8	178.2 (3)
C6—C7—C8—C14	−163.2 (3)	C12—C13—C14—C15	166.8 (3)
C6—C7—C8—C9	−41.8 (4)	C18—C13—C14—C15	−70.8 (4)
C14—C8—C9—C11	−49.4 (3)	C17—C13—C14—C15	45.5 (3)
C7—C8—C9—C11	−172.1 (3)	C8—C14—C15—C16	−165.9 (3)
C14—C8—C9—C10	−178.2 (3)	C13—C14—C15—C16	−35.7 (4)
C7—C8—C9—C10	59.2 (3)	C14—C15—C16—C17	11.1 (4)
C6—C5—C10—C1	133.6 (4)	C15—C16—C17—C20	142.5 (3)
C4—C5—C10—C1	−47.1 (4)	C15—C16—C17—C13	16.6 (4)
C6—C5—C10—C19	−107.5 (4)	C14—C13—C17—C20	−162.4 (3)
C4—C5—C10—C19	71.9 (3)	C12—C13—C17—C20	82.3 (4)
C6—C5—C10—C9	14.9 (4)	C18—C13—C17—C20	−43.4 (4)
C4—C5—C10—C9	−165.7 (3)	C14—C13—C17—C16	−37.4 (3)
C2—C1—C10—C5	49.6 (4)	C12—C13—C17—C16	−152.7 (3)
C2—C1—C10—C19	−68.4 (4)	C18—C13—C17—C16	81.6 (4)
C2—C1—C10—C9	169.1 (3)	C16—C17—C20—O2	−26.9 (5)
C8—C9—C10—C5	−44.6 (3)	C13—C17—C20—O2	93.0 (5)
C11—C9—C10—C5	−173.2 (3)	C16—C17—C20—N1	156.3 (3)
C8—C9—C10—C1	−163.0 (3)	C13—C17—C20—N1	−83.7 (4)
C11—C9—C10—C1	68.4 (3)	O2—C20—N1—C21	1.8 (6)
C8—C9—C10—C19	75.7 (3)	C17—C20—N1—C21	178.5 (4)
C11—C9—C10—C19	−52.9 (4)	C20—N1—C21—C23	178.2 (4)
C8—C9—C11—C12	50.1 (4)	C20—N1—C21—C24	59.4 (5)
C10—C9—C11—C12	179.0 (3)	C20—N1—C21—C22	−62.2 (6)
C9—C11—C12—C13	−53.5 (4)	O1—C3—C2—C1	−179.3 (3)
C11—C12—C13—C14	55.9 (4)	C4—C3—C2—C1	57.7 (4)
C11—C12—C13—C18	−68.1 (4)	C10—C1—C2—C3	−57.1 (4)
C11—C12—C13—C17	166.7 (3)		

### Hydrogen-bond geometry (Å, °)

**Table:**

*D*—H···*A*	*D*—H	H···*A*	*D*···*A*	*D*—H···*A*
O1—H1OA···O1^i^	0.82 (1)	1.99 (4)	2.783 (6)	161 (10)
O1W—H1W···O1W^i^	0.85 (1)	2.26 (6)	2.798 (9)	121 (6)
O1—H1OB···O1W^ii^	0.82 (1)	2.31 (9)	2.756 (5)	115 (8)
O1W—H2WA···O1^iii^	0.85 (1)	1.93 (6)	2.756 (5)	163 (20)

Symmetry codes: (i) −*x*+1, *y*, −*z*+1; (ii) −*x*+1/2, *y*−1/2, −*z*+1; (iii) −*x*+1/2, *y*+1/2, −*z*+1.
